# Supplementary figures and images for: JNK and Yorkie drive tumor malignancy by inducing L-amino acid transporter 1 in Drosophila
Source: PLoS Genet. 2021 Nov 15;17(11):e1009893. doi: 10.1371/journal.pgen.1009893 (PMC8629376; doi:10.1371/journal.pgen.1009893)

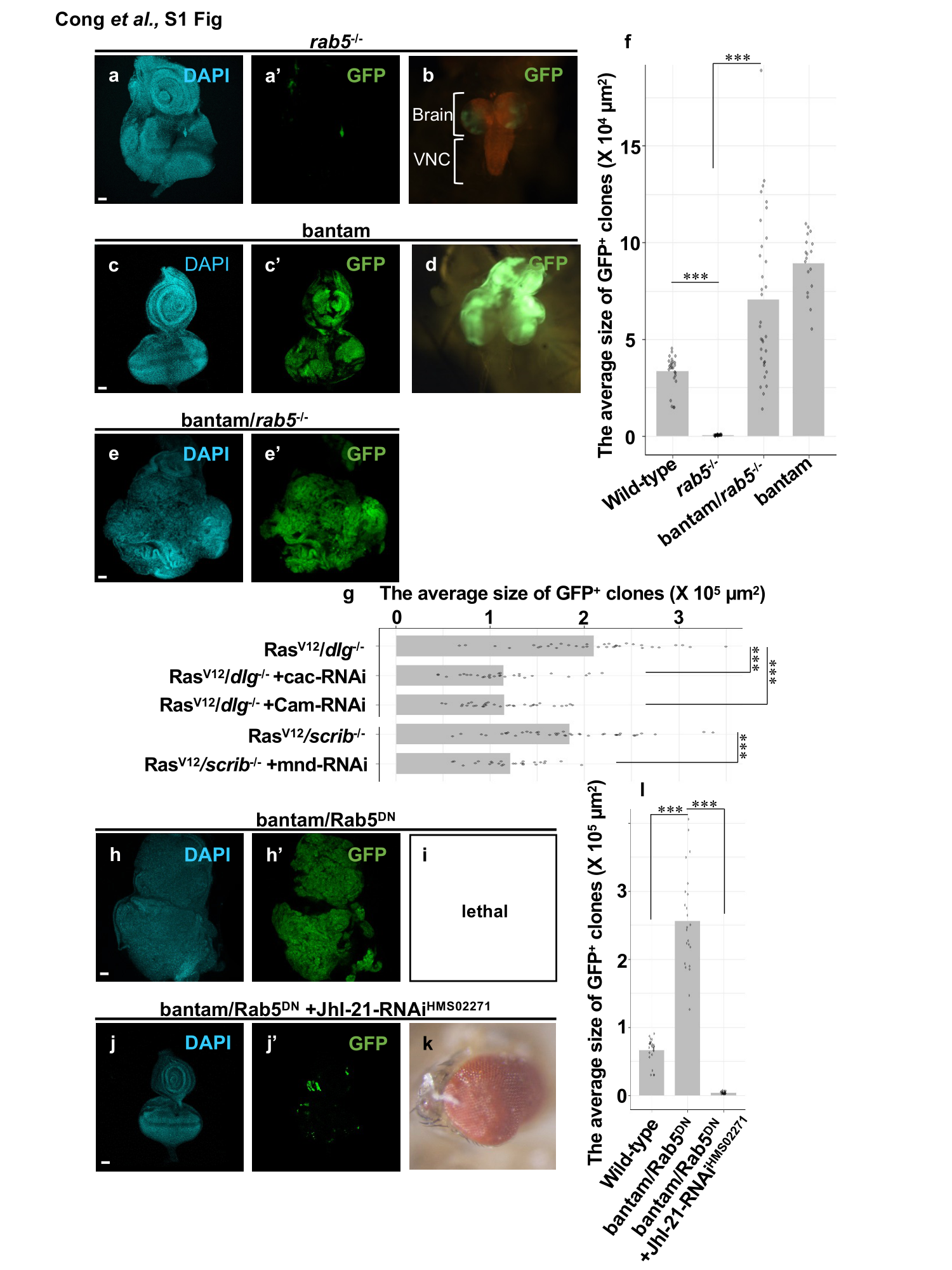

Supplement: S1 Fig — (a, c, e, h, i, j and k) Eye-antennal disc bearing GFP-labeled rab5-/- (a), bantam (c), bantam/rab5-/- (e), bantam/Rab5DN (h and i), and bantam/Rab5DN +JhI-21-RNAi (HMS02271) (j and k) clones are shown. Cell nuclei were stained with DAPI. (b and d) Images of cephalic complexes, which include brain and the ventral nerve cord (VNC). (f) The average size of Wild-type (n = 26), rab5-/- (n = 40), bantam/rab5-/- (n = 33), and bantam (n = 18) clones were measured by ImageJ. (g) The average size of clones of GFP-labeled RasV12/dlg-/- (n = 35), RasV12/dlg-/- +cac-RNAi (n = 29), RasV12/dlg-/- +Cam-RNAi (n = 32), RasV12/scrib-/- (n = 41), and RasV12/scrib-/- +mnd-RNAi (n = 25) clones were measured by ImageJ. (l) The average size of Wild-type (n = 26), bantam/Rab5DN (n = 23), and bantam/Rab5DN +JhI-21RNAi (HMS02271) (n = 25) clones were measured by ImageJ. Scale bars, 50 μm. See S3 Table for details of statistical analyses. (TIF) [file pgen.1009893.s001.tif]

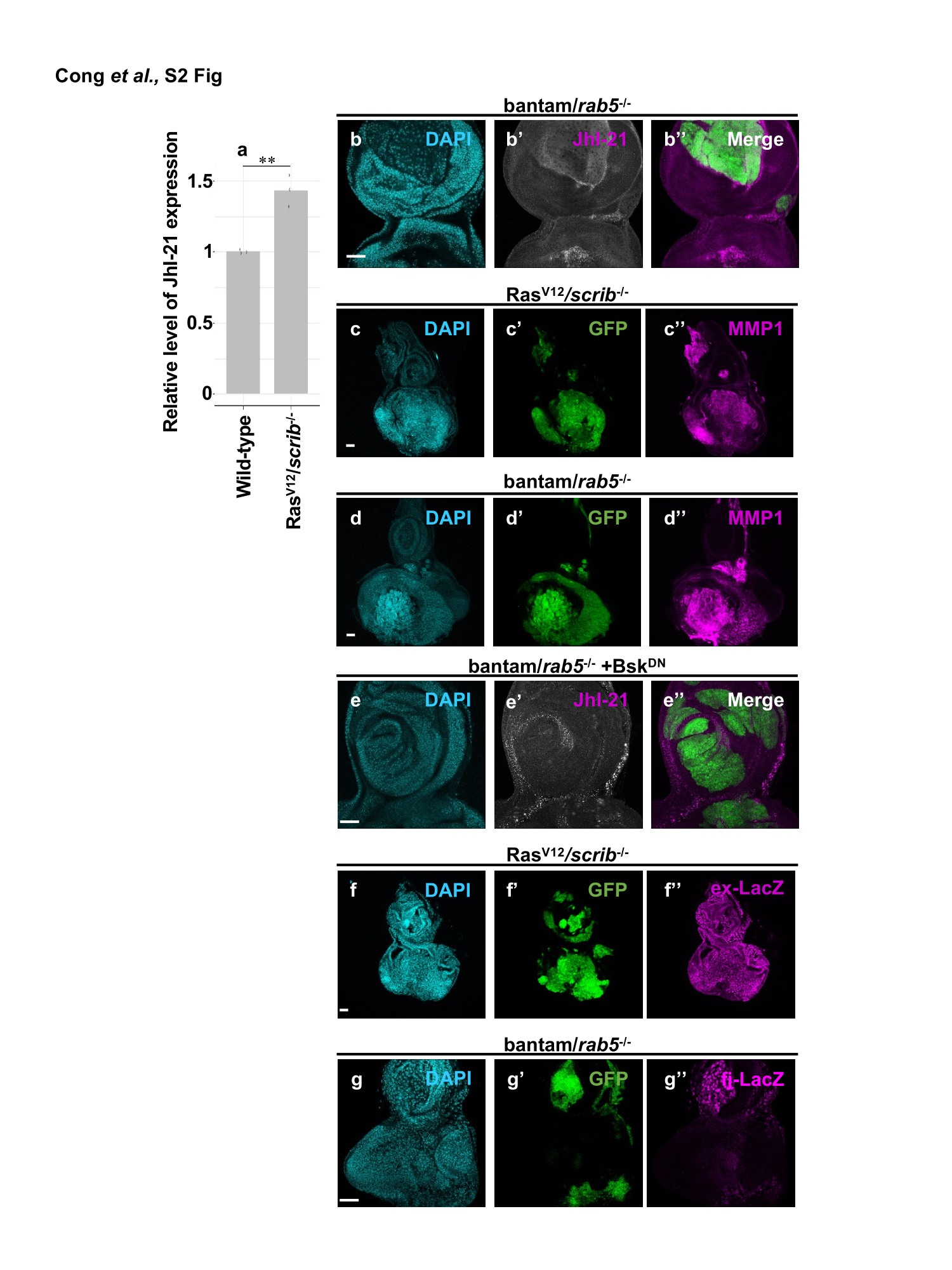

Supplement: S2 Fig — (a) Quantitative RT-PCR revealed equal JhI-21 mRNA expression levels among genotypes. Statistics analyses see S3 Table. (b, d and g) GFP-labeled bantam/rab5-/- clones were induced in eye-antennal discs and were stained with anti-JhI-21 antibody (b), anti-MMP1 (a JNK target) antibody (d) or anti-β-galactosidase antibody to detect Yki-target fj-lacZ expression (g). (c and f) GFP-labeled RasV12/scrib-/- clones were induced in eye-antennal discs and were stained with anti-MMP1 antibody (c) or anti-β-galactosidase antibody to detect Yki-target ex-lacZ expression (f). (e) GFP-labeled bantam/rab5-/- + BskDN clones were induced in eye-antennal discs and were stained with anti-JhI-21 antibody. Cell nuclei were stained with DAPI. Scale bars, 50 μm. (TIF) [file pgen.1009893.s002.tif]

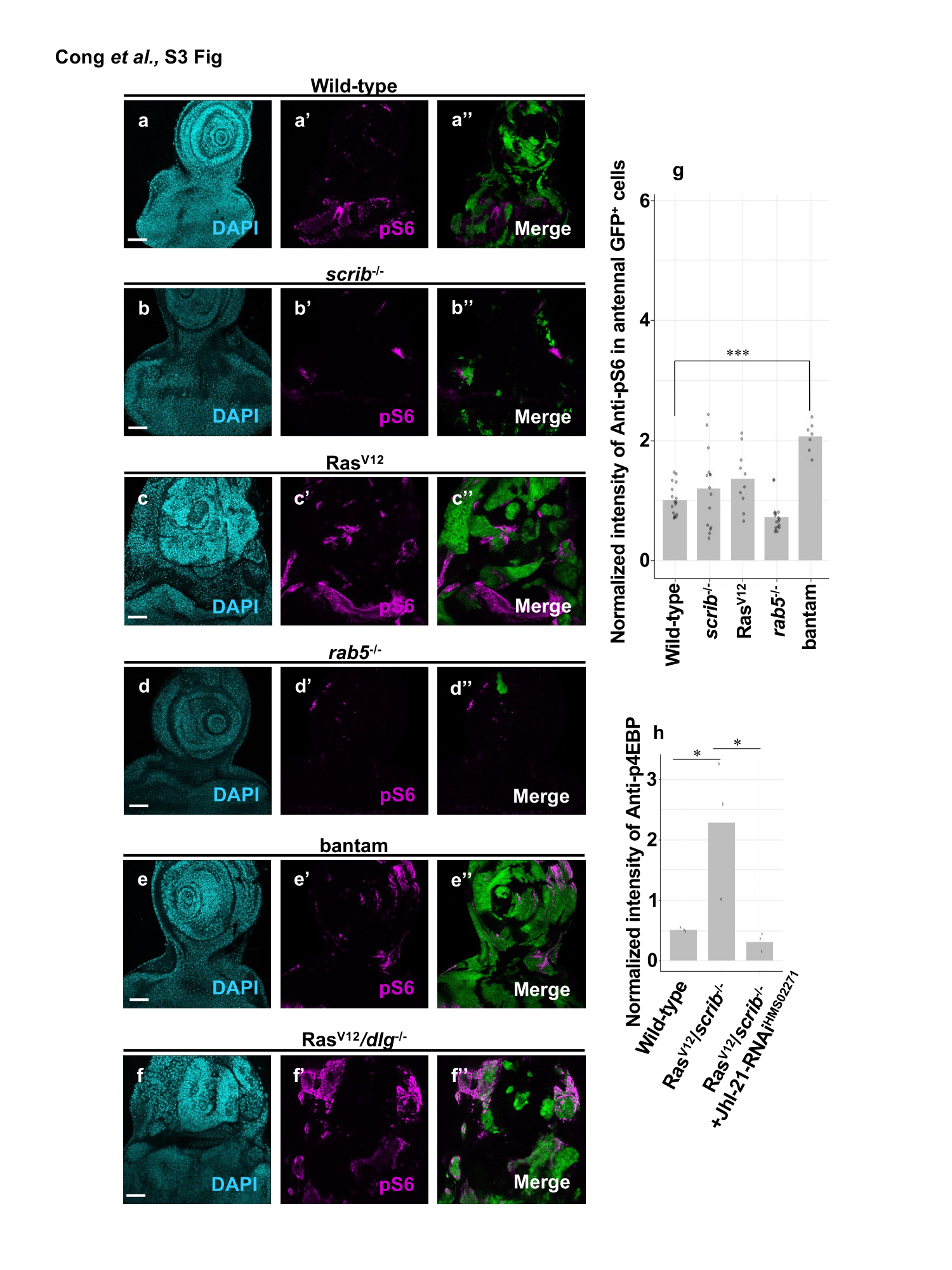

Supplement: S3 Fig — (a, b, c, d e and f) GFP-labeled Wild-type (a), scrib-/- (b), RasV12 (c), rab5-/- (d), bantam (e), and RasV12/dlg-/- (f) clones were induced in eye-antennal disc and were stained with anti-pS6 antibody. (g) The averaged normalized intensity of anti-pS6 in antennal GFP+ cells in each genotype: Wild-type (n = 17), scrib-/- (n = 15), RasV12 (n = 10), rab5-/- (n = 18), bantam (n = 7). (h) The average normalized intensity of anti-p4EBP in antennal discs in each genotype: Wild-type (n = 3), RasV12/scrib-/- (n = 3), RasV12/scrib-/- +JhI-21-RNAi (HMS02271) (n = 3) and normalized by α-Tubulin. The intensity was measured by ImageJ. Cell nuclei were stained with DAPI. Scale bars, 50 μm. See S3 Table for details of statistical analyses. (TIF) [file pgen.1009893.s003.tif]

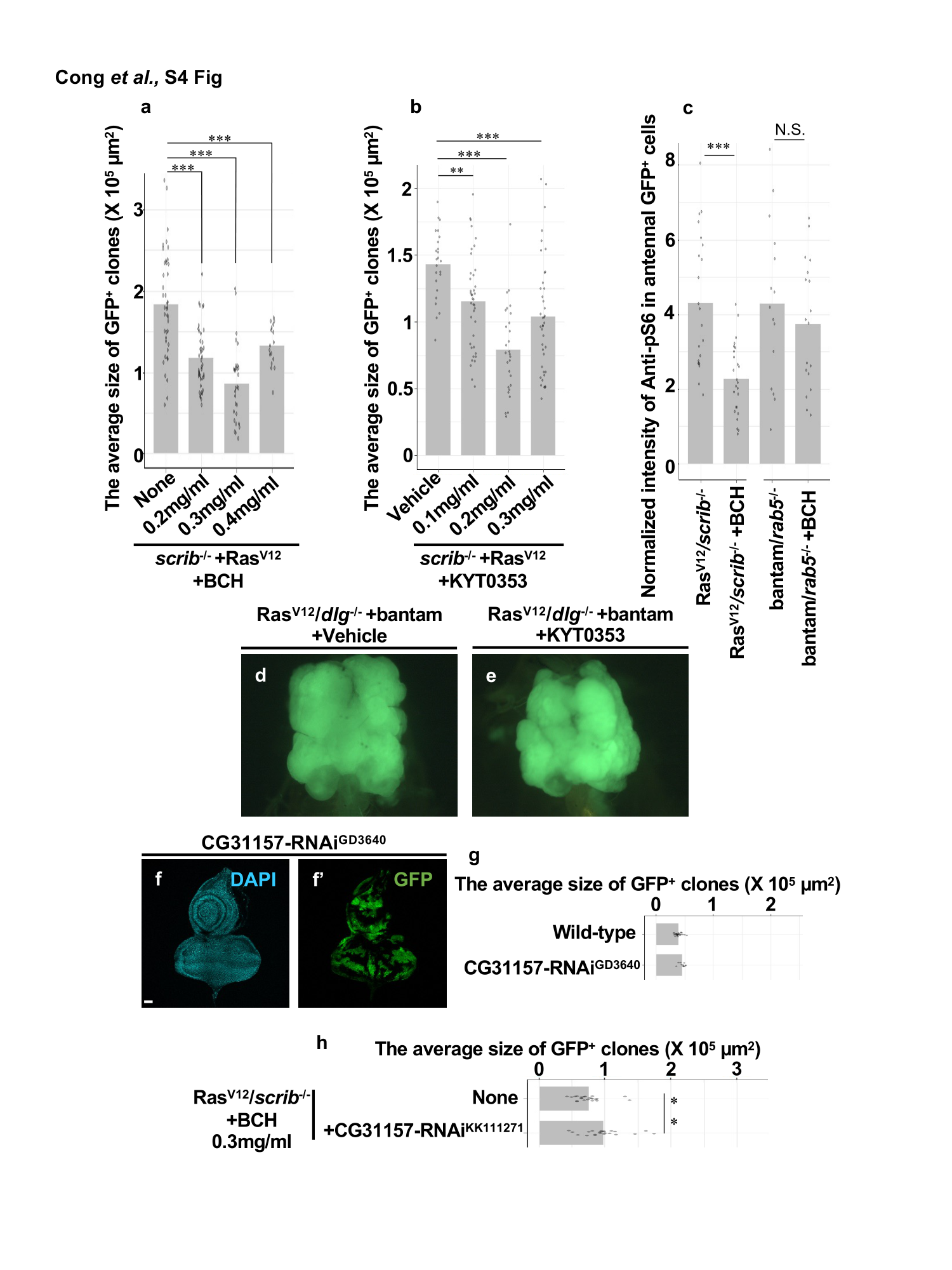

Supplement: S4 Fig — (a and b) Quantification of average sizes of GFP+ clones of RasV12/scrib-/- (day7) after larval administration of different dosages BCH (a) or KYT0353 (b). (a) RasV12/scrib-/- (None, n = 41) RasV12/scrib-/- (0.2mg/ml, n = 42) RasV12/scrib-/- (0.3mg/ml, n = 68) RasV12/scrib-/- (0.4mg/ml, n = 17). (b) RasV12/scrib-/- (Vehicle, n = 24) RasV12/scrib-/- (0.1mg/ml, n = 40) RasV12/scrib-/- (0.2mg/ml, n = 28) RasV12/scrib-/- (0.3mg/ml, n = 40). (c) The averaged normalized intensity of anti-pS6 in antennal GFP+ cells in each genotype: RasV12/scrib-/- (n = 22), RasV12/scrib-/- +BCH (0.3mg/ml) (n = 25), bantam/rab5-/- (n = 15), bantam/rab5-/- +BCH (0.3mg/ml) (n = 18). The intensity was measured by ImageJ. (d and e) Images of cephalic complexes, which include brain and the VNC. GFP-labeled RasV12/dlg-/- + bantam clones were induced in eye-antennal disc and the larvae were administrated with Vehicle or KYT0353 (0.2mg/ml) (images are cephalic complexes at day 7). (f) GFP-labeled CG31157-RNAi (GD3640)-expressing clones were induced in eye-antennal disc. Cell nuclei were stained with DAPI. Scale bars, 50 μm. (g) The average size of Wild-type (n = 26) and CG31157-RNAi (GD3640) (n = 11) clones were measured by ImageJ. (h) Quantification of average sizes of GFP+ clones of RasV12/scrib-/- (day7, n = 23) or RasV12/scrib-/- +CG31157-RNAi (KK111271) (day7, n = 23) after larval administration of BCH. See S3 Table for details of statistical analyses. (TIF) [file pgen.1009893.s004.tif]
