# Supplementary material for: JNK and Yorkie drive tumor malignancy by inducing L-amino acid transporter 1 in Drosophila
Source: PLoS Genet. 2021 Nov 15;17(11):e1009893. doi: 10.1371/journal.pgen.1009893 (PMC8629376; doi:10.1371/journal.pgen.1009893)
Supplement: S1 Text — (DOCX) [file pgen.1009893.s008.docx]

**Detailed genotypes used in each figure**

Fig 1

*eyFLP1/+ or Y; Act>y+>Gal4, UAS-GFP/+; FRT82B, Tub-Gal80/FRT82B* (a, i and m), *eyFLP1/+ or Y; Act>y+>Gal4, UAS-GFP/+; FRT82B, Tub-Gal80/UAS-Ras^V12^, FRT82B, scrib^1^* (b, g and h), *eyFLP1/+ or Y; FRT40A, Tub-Gal80/FRT40A rab5^LL00467^; Act>y+>Gal4, UAS-GFP/UAS-bantam* (c), *eyFLP1/+ or Y; Act>y+>Gal4, UAS-GFP/UAS-JhI-21-RNAi; FRT82B, Tub-Gal80/UAS-Ras^V12^, FRT82B, scrib^1^* (j, k, l), *eyFLP1/+ or Y; Act>y+>Gal4, UAS-GFP/UAS-JhI-21-RNAi; FRT82B, Tub-Gal80/FRT82B* (n).

Fig 2

*eyFLP1/+ or Y; Act>y+>Gal4, UAS-GFP/+; FRT82B, Tub-Gal80/UAS-Ras^V12^, FRT82B, scrib^1^* (a), *eyFLP1/UAS-Bsk^DN^; Act>y+>Gal4, UAS-GFP/+; FRT82B, Tub-Gal80/UAS-Ras^V12^, FRT82B, scrib^1^* (b), *eyFLP1/+ or Y; FRT40A, Tub-Gal80/FRT40A, UAS-eiger^W^; Act>y+>Gal4, UAS-GFP/+* (c), *eyFLP1/+ or Y; Act>y+>Gal4, UAS-GFP/UAS-wts; FRT82B, Tub-Gal80/UAS-Ras^V12^, FRT82B, scrib^1^* (d), *eyFLP1/+ or Y; FRT40A, Tub-Gal80/FRT40A, UAS-Yki^S168A^; Act>y+>Gal4, UAS-GFP/+* (e), and *eyFLP1/+ or Y; FRT40A, Tub-Gal80/FRT40A UAS-eiger^12^; Act>y+>Gal4, UAS-GFP/UAS-Yki* (f).

Fig 3

*eyFLP1/+ or Y; Act>y+>Gal4, UAS-GFP/+; FRT82B, Tub-Gal80/UAS-Ras^V12^, FRT82B, scrib^1^* (a and g), *eyFLP1; Act>y+>Gal4, UAS-GFP/+; FRT82B, Tub-Gal80/UAS-bantam, Rab5^DN^, FRT82B* (b), *eyFLP1/+ or Y; Act>y+>Gal4, UAS-GFP/UAS-JhI-21-RNAi; FRT82B, Tub-Gal80/UAS-Ras^V12^, FRT82B, scrib^1^* (c and g), *eyFLP1; Act>y+>Gal4, UAS-GFP/UAS-JhI-21-RNAi; FRT82B, Tub-Gal80/UAS-bantam, Rab5^DN^, FRT82B* (d), *FRT19A, Tub-Gal80/FRT19A, dlg^m52^; eyFRP5, Act>y+>Gal4, UAS-GFP/UAS-Ras^V12^; +/UAS-Rheb-RNAi* (e and i), *eyFLP1/+ or Y; Act>y+>Gal4, UAS-GFP/+; FRT82B, Tub-Gal80/FRT82B* (f and g), *eyFLP1/+ or Y; FRT40A, Tub-Gal80/FRT40A; Act>y+>Gal4, UAS-GFP/UAS-Rheb-RNAi* (h), *FRT19A, Tub-Gal80/FRT19A, dlg^m52^; eyFRP5, Act>y+>Gal4, UAS-GFP/UAS-Ras^V12^; +/sb* (j).

Fig 4

*eyFLP1/+ or Y; FRT40A, Tub-Gal80/FRT40A; Act>y+>Gal4, UAS-GFP/+* (a and b), *eyFLP1/+ or Y; Act>y+>Gal4, UAS-GFP/+; FRT82B, Tub-Gal80/UAS-Ras^V12^, FRT82B, scrib^1^* (a and b), *eyFLP1/+ or Y; FRT40A, Tub-Gal80/FRT40A rab5^LL00467^; Act>y+>Gal4, UAS-GFP/UAS-bantam* (a, b, e and j), *FRT19A, Tub-Gal80/FRT19A, dlg^m52^; eyFRP5, Act>y+>Gal4, UAS-GFP/UAS-Ras^V12^; +/sb* (c, d and f), *FRT19A, Tub-Gal80/FRT19A, dlg^m52^; eyFRP5, Act>y+>Gal4, UAS-GFP/UAS-Ras^V12^; +/UAS-bantam* (c and d), *FRT19A, Tub-Gal80/FRT19A, dlg^m52^; eyFRP5, Act>y+>Gal4, UAS-GFP/UAS-Ras^V12^; +/UAS-CG31157-RNAi^GD3640^* (g and h), *eyFLP1/UAS-CG31157; FRT40A, Tub-Gal80/FRT40A rab5^LL00467^; Act>y+>Gal4, UAS-GFP/UAS-bantam* (j).

S1 Fig

*eyFLP1/+ or Y; FRT40A, Tub-Gal80/FRT40A, rab5^LL00467^; Act>y+>Gal4, UAS-GFP/+* (a and b), *eyFLP1/+ or Y; FRT40A, Tub-Gal80/FRT40A; Act>y+>Gal4, UAS-GFP/UAS-bantam* (c and d), *eyFLP1/+ or Y; FRT40A, Tub-Gal80/FRT40A, rab5^LL00467^; Act>y+>Gal4, UAS-GFP/UAS-bantam* (e). (g) *FRT19A, Tub-Gal80/FRT19A, dlg^m52^; eyFRP5, Act>y+>Gal4, UAS-GFP/UAS-Ras^V12^; +/sb*, *FRT19A, Tub-Gal80/FRT19A, dlg^m52^; eyFRP5, Act>y+>Gal4, UAS-GFP/UAS-Ras^V12^; +/UAS-cac-RNAi*, *FRT19A, Tub-Gal80/FRT19A, dlg^m52^; eyFRP5, Act>y+>Gal4, UAS-GFP/UAS-Ras^V12^; +/UAS-Cam-RNAi*, *eyFLP1/+ or Y; Act>y+>Gal4, UAS-GFP/+; FRT82B, Tub-Gal80/UAS-Ras^V12^, FRT82B, scrib^1^*, and *eyFLP1/+ or Y; Act>y+>Gal4, UAS-GFP/UAS-mnd-RNAi; FRT82B, Tub-Gal80/UAS-Ras^V12^, FRT82B, scrib^1^*. *eyFLP1/+ or Y; FRT40A, Tub-Gal80/FRT40A; Act>y+>Gal4, UAS-GFP/UAS-Rab5^DN^, UAS-bantam* (h and i), *eyFLP1/+ or Y; FRT40A, Tub-Gal80/FRT40A, UAS-JhI-21-RNAi; Act>y+>Gal4, UAS-GFP/UAS-Rab5^DN^, UAS-bantam* (j and k).

S2 Fig

*eyFLP1/+ or Y; Act>y+>Gal4, UAS-GFP/+; FRT82B, Tub-Gal80/FRT82B* (a), *eyFLP1/+ or Y; FRT40A, Tub-Gal80/FRT40A, rab5^LL00467^; Act>y+>Gal4, UAS-GFP/UAS-bantam* (b and d), *eyFLP1/+ or Y; Act>y+>Gal4, UAS-GFP/+; FRT82B, Tub-Gal80/UAS-Ras^V12^, FRT82B, scrib^1^* (a and c), *eyFLP1/USA-Bsk^DN^; FRT40A, Tub-Gal80/FRT40A, rab5^LL00467^; Act>y+>Gal4, UAS-GFP/UAS-bantam* (e), *eyFLP1/+ or Y; Act>y+>Gal4, UAS-GFP/ex-LacZ; FRT82B, Tub-Gal80/UAS-Ras^V12^, FRT82B, scrib^1^* (f), *eyFLP1/+ or Y; fj-LacZ, FRT40A, Tub-Gal80/FRT40A, rab5^LL00467^; Act>y+>Gal4, UAS-GFP/UAS-bantam* (g).

S3 Fig

*eyFLP1/+ or Y; Act>y+>Gal4, UAS-GFP/+; FRT82B, Tub-Gal80/FRT82B* (a), *eyFLP1/+ or Y; Act>y+>Gal4, UAS-GFP/+; FRT82B, Tub-Gal80/FRT82B, scrib^1^* (b), *eyFLP1/+ or Y; Act>y+>Gal4, UAS-GFP/+; FRT82B, Tub-Gal80/UAS-Ras^V12^, FRT82B* (c), *eyFLP1/+ or Y; FRT40A, Tub-Gal80/FRT40A, rab5^LL00467^; Act>y+>Gal4, UAS-GFP/+* (d), *eyFLP1/+ or Y; FRT40A, Tub-Gal80/FRT40A; Act>y+>Gal4, UAS-GFP/UAS-bantam* (e), *FRT19A, Tub-Gal80/FRT19A, dlg^m52^; eyFRP5, Act>y+>Gal4, UAS-GFP/UAS-Ras^V12^; +/sb* (f).

S4 Fig

*eyFLP1/+ or Y; Act>y+>Gal4, UAS-GFP/+; FRT82B, Tub-Gal80/UAS-Ras^V12^, FRT82B, scrib^1^*(a, b and c), *eyFLP1/+ or Y; FRT40A, Tub-Gal80/FRT40A, rab5^LL00467^; Act>y+>Gal4, UAS-GFP/UAS-bantam* (c), *FRT19A, Tub-Gal80/FRT19A, dlg^m52^; eyFRP5, Act>y+>Gal4, UAS-GFP/UAS-Ras^V12^; UAS-bantam/+* (d and e), *eyFLP1/+ or Y; FRT40A, Tub-Gal80/FRT40A; Act>y+>Gal4, UAS-GFP/UAS-CG31157-RNAi* (f), *eyFLP1/+ or Y; Act>y+>Gal4, UAS-GFP/UAS-CG31157-RNAi; FRT82B, Tub-Gal80/UAS-Ras^V12^, FRT82B, scrib^1^*(h).
