# Supplementary material for: JNK and Yorkie drive tumor malignancy by inducing L-amino acid transporter 1 in Drosophila
Source: PLoS Genet. 2021 Nov 15;17(11):e1009893. doi: 10.1371/journal.pgen.1009893 (PMC8629376; doi:10.1371/journal.pgen.1009893)
Supplement: S2 Text — (DOCX) [file pgen.1009893.s009.docx]

**Supplementary methods**

**Supplementary Fly strains and genetics**

Fly stocks were cultured at room temperature or 25°C on standard fly food. Fly stocks used were: *eyFLP1; TubGal80, FRT40A; Act> y+ >Gal4, UAS-GFP* (40A tester)；*eyFLP1; Act> y+ >Gal4, UAS-GFP; FRT82B, TubGal80* (82B tester)；*FRT19A, Tub-Gal80; eyFRP5, Act>y+>Gal4, UAS-GFP; sb/TM6B* (19A tester).

Additional strains used are as follows: UAS-Ras^V12^ (BL4847, BL5788), UAS-bantam (BL60672), UAS-JhI-21-RNAi (BL41706), UAS-mnd-RNAi (BL62207), UAS-Rab5^DN^ (BL42704), UAS-Cam-RNAi (BL34609), UAS-cac-RNAi (4236R-1, NIG-FLY), *rab5*^LL00467^ (DGRC), UAS-CG31157-RNAi (VDRC30296, VDRC102256), *scrib*^1^ (gift from D. Bilder), *dlg*^m52^ (gift from N. Perrimon), UAS-Bsk^DN^ (gift from T. Adachi-Yamada).

**Supplementary Immunohistochemistry**

Third instar larvae were dissected in PBS and fixed in 4% paraformaldehyde in PBS for 25 min. Following 4 washes with PBT (PBS+0.1% Triton X-100), samples were blocked with PBT + 5% Normal Donkey Serum for 30 min before incubation with primary antibodies in PBT + 5% Normal Donkey Serum overnight. Secondary antibodies were incubated for 2 hours at RT before mounting in DAPI-containing SlowFade Gold Antifade Reagent (Molecular Probes). Primary antibodies used: mouse Mmp1 monoclonal antibody (1:100 from 1:1:1 cocktail of 3A6B4, 3B8D12, and 5H7B11; DSHB), 1:100 rabbit anti-JhI-21 (gift from Y. Grosjean; in Can Get Signal Immunoreaction Enhancer Solution A), rabbit anti-Phospho-S6 (1:400; gift from J. Chung), 1:200 chicken galactosidase antibody (abcam, #ab9361). Secondary antibodies used: anti-rabbit, chicken or mouse Alexafluor 546 or 647 (1:250). Images were taken with a Leica SP5.

**Supplementary FACS and mRNA-seq analyses**

Third instar larvae were dissected in calcium-free PBS and eye-antennal discs were collected in calcium-free PBS. Eye-antennal discs were incubated in 200 μl of 10x TrypLE Select (Life Technologies) at 37^o^C for 20 min. Enzyme reaction was stopped by 5% FBS Schneider’s medium and cells were dissociated by pipetting 2-3 times with a 1,000 μl pipet tip. 100,000 GFP-positive cells were sorted by a BD FACS Aria II cell sorter, and total 15 ng RNA was isolated using NucleoSpin RNA XS Kit (TaKaRa). RNA integrity was determined with an Agilent 2100 Bioanalyzer, and then mRNAs were purified from 10ng of the total RNAs by NEBNext Poly(A) mRNA Magnetic Isolation Module (E7490S) and Strand-specific Libraries for mRNA-seq were prepared by using the NEBNext Ultra II Directional RNA Library Prep Kit for Illumina (E7760S) with 14 PCR cycles. RNA-seq was performed with NextSeq 500/550 High output Kit v2.5 (75 Cycles) on a NextSeq 500 (illumine) to obtain single-end reads at a length of 76 bases. For each experiment, three biological replicates were analyzed.

**Supplementary Quantitative RT-PCR**

Third instar larvae were dissected in PBS and eye-antennal discs were collected in PBS. RNA from 20~30 discs per genotype was isolated using NucleoSpin RNA XS Kit (TaKaRa). RNA samples were reverse transcribed using the SuperScript IV reverse transcriptase (Thermo Fisher Scientific). cDNA samples were prepared by SYBR Green reagent (TOYOBO). Real-time PCR was performed using The StepOnePlus Real-Time PCR System.

Primers:

*JhI-21* (TTGTTTACCACGGCGAAATAG/CTTTGTGACGGAGGAGCTACA); control gene: *RpL32* (CCAAGATCGTGAAGAAGCG/GTTGGGCATCAGATACTGTC).
