# Supplementary material for: JNK and Yorkie drive tumor malignancy by inducing L-amino acid transporter 1 in Drosophila
Source: PLoS Genet. 2021 Nov 15;17(11):e1009893. doi: 10.1371/journal.pgen.1009893 (PMC8629376; doi:10.1371/journal.pgen.1009893)
Supplement: Attachment — Submitted filename: Responses to Reviewers.ver.2.pdf [file pgen.1009893.s010.pdf]

We would like to thank the reviewers for excellent comments, which have led us to improve our manuscript substantially. Modifications are indicated in red in the revised manuscript.

Response to Reviewers' Comments:

Reviewer #1 (Comments to the Author):

In this study, to characterize the common oncogenesis pathways, Cong et al compared differentially-expressed genes between RasV12/scrib-/- and bantam/rab5-/- larval disc tumors and identified JhI-21, a fly homolog of LAT1 that senses Leucine availability, as a crucial regulator for the growth of both of them. They further found that JhI-21 expression is commonly increased by JNK and yki signaling pathways to increase mTOR activity and promote growth in these two tumors. However, when authors fed larvae LAT1 inhibitors to try to inhibit Jhl-21 activity, they interestingly uncovered that the growth of only RasV12/scrib-/- tumors, but not bantam/rab5-/- tumors, was suppressed. Finally, combining RNA-seq and RNAi, they indicated the molecular mechanism(s) of drug resistance in bantam/rab5-/- tumors, whereby bantam suppresses the expression of CG31157, a TMEM135-like gene, to diminish effects of LAT1 inhibitors in the larval disc tumors.

Given the known fact that LAT1/2 mediates Leucine uptake, mTOR activation and tumor progression in mammals, this work provides novel findings of differential drug effects in two different tumors, mimicking genetic complexity and heterogenous nature of mammalian tumors. The authors also preliminarily indicated that bantam/CG31157 axis could be the key regulator of drug resistance, which could be possibly translated into future human cancer treatment. However, the current results of Leucine sensing, Jhi-21 inhibition, and CG31157 functions still are quite obscure. I recommend a major revision prior to the publication in Plos Genetics.

1. Multiple RNAi lines or mutants of JhI-21 and CG31157 should be used to exclude the off-target effects. In this study, only a single RNAi line for either JhI-21 or CG31157 was not sufficient to conclude the loss-of-function effects.

Response:
We thank the reviewer for the comment. Following the reviewer's suggestion, we have performed additional experiments using other JhI-21-RNAi and CG31157-RNAi lines and found that different JhI-21-RNAi or CG31157-RNAi lines showed the same result (please see revised **Figure 1o** and **Supplemental Figure S4h**). We have now included these new data in the revised manuscript as follows:

(page 6)
"The strongest effect was observed by JhI-21 knockdown (using two different

RNAi lines HMS02271 and KK112996; Fig. 1o).”

(page 9)

“A similar result was obtained by using a different RNAi line for *CG31157* (KK111271) (Supplementary Figure S4h)”

2. As indicated in Grosjean’s work, a poor medium supplied with Leucine is recommended to validate Leucine/JhI-21 axis in tumor growth regulation.

Response:

We thank the reviewer for the comment. Following the reviewer’s suggestion, we have performed additional experiments as follows. We fed larvae bearing $Ras^{V12}/scrib^{-/-}$ tumors in the eye-antennal discs with low-yeast diet (“poor medium”) with or without L-leucine. In the case of normal diet, many 3rd instar $Ras^{V12}/scrib^{-/-}$ larvae crawled out of the food at day5. However, when the larvae were fed with low-yeast diet, very few 3rd instar $Ras^{V12}/scrib^{-/-}$ larvae still resided in the food at day14. When the larvae were fed with low-yeast diet plus L-leucine, some 3rd instar $Ras^{V12}/scrib^{-/-}$ larvae crawled out of the food at day14. These data intriguingly suggest that L-leucine is essential for normal development and survival of the larvae. However, we found that there was no significant difference in the tumor size with or without L-leucine (please see Figure R1 for the reviewer, below). Given that the amount of L-leucine in the food affects the growth and development of the larvae bearing tumors, it might be difficult to evaluate the role of Leucine in tumorigenesis in this experimental setting.

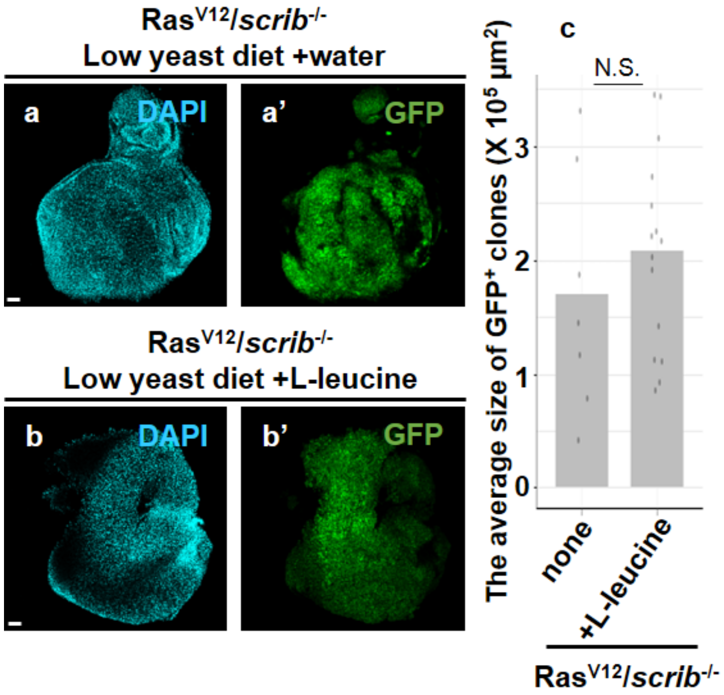

Figure R1 There was no significant difference in tumor size with or without L-leucine in low-yeast diet.
(a and b) GFP-labeled Ras^{V12}/*scrib*$^{-/-}$ clones were induced in eye-antennal discs in the larvae fed with low-yeast diet (2g/l) without (a) or with L-leucine (20mM) at day 14. (c) Average sizes of Ras^{V12}/*scrib*$^{-/-}$ (n=7), and Ras^{V12}/*scrib*$^{-/-}$ +L-leucine (n=15) clones were measured by ImageJ. Student's t-test, N.S P>0.05, *P<0.05, **P<0.01 and ***P<0.001. Scale bars, 50 µm.
Genotype: *eyFLP1/+ or Y; Act>y+>Gal4, UAS-GFP/+; FRT82B, Tub-Gal80/UAS-Ras*^{V12}*, FRT82B, scrib*^{1} (a and b).

3. Although BCH has been previously shown to slow down Leucine uptake, the effects of Kyt-0353/JPH203 were not well evaluated in Drosophila. Because the drug concentrations in the fly food are so high (0.3 mg/mL BCH equals 2 mM, 0.2 mg/mL KYT-0353 equals 4 mM), different dosages should be tested.
On the other hand, the larvae were fed inhibitors simultaneously with tumor induction. The authors actually compared pS6 levels in the normal cells and well-expanded tumors, but not tumor responses to inhibitors. Therefore, the short-term feeding of drugs should be performed. For example, larvae are normally cultured for 6-9 days to develop disc tumors and then are fed drugs for 12 or 24 hours to evaluate pS6 levels in the tumors. In vitro assays that treat isolated disc tumors with Leucine and different drugs are also recommended.

Response:
As for the former comment, we have tested different dosages of BCH and KYT-0353 (please see revised **Supplemental Figure S4a** and **S4b**). We found that 0.3 mg/mL BCH and 0.2 mg/mL KYT-0353 were the most effective dosages for the tumor suppression. The effective drug concentrations in the fly food for feeding larvae are generally very high compared to those for cell cultures, for instance 5 mM chloroquine for autophagy inhibition (Katheder et al., Nature, 2017) and 5 mM metformin for mitochondrial complex I inhibition (Sanaki et al., Dev Cell, 2020). We have now included these new data in the revised manuscript as follows:

(page 8)
"Notably, we found that feeding BCH or KYT0353 to larvae bearing Ras^{V12}/*scrib*$^{-/-}$ tumors in the eye-antennal discs significantly reduced tumor growth, while these drugs did not affect growth of wild-type clones (Fig. 4a, 4b, Supplementary Figure S4a and S4b)."

As for the latter comment, the larvae stop feeding food around the time that they form tumors in the eye-antennal discs. Therefore, it is technically difficult to examine the effect of short-time drug feeding on tumors after they generate tumors. Therefore, as suggested by the reviewer, we tried to perform *in vitro* assays to evaluate pS6 levels in the tumors. As a control experiment, we found that pS6 staining was elevated at 0 hr in Ras^{V12}/*scrib*$^{-/-}$ tumors (please see Figure R2a, below). However, after 8 hr *in vitro*

cultures of the Ras^{V12}/*scrib*$^{-/-}$ tumors, we could not detect pS6 staining in Ras^{V12}/*scrib*$^{-/-}$ tumors (please see Figure R2b, below). This culture condition works for live-imaging of imaginal discs to evaluate cell proliferation and cell death (Akai et al., PLOS Genetics, 2021). These data suggest that culturing tumors *in vitro* requires unidentified special experimental conditions. We have tried to optimize the condition but have realized that it would take us more than 6 months or even a year to establish the condition for tumor culture *in vitro* and thus would like to address this issue in our future studies. Again, we thank the reviewer for the interesting comment.

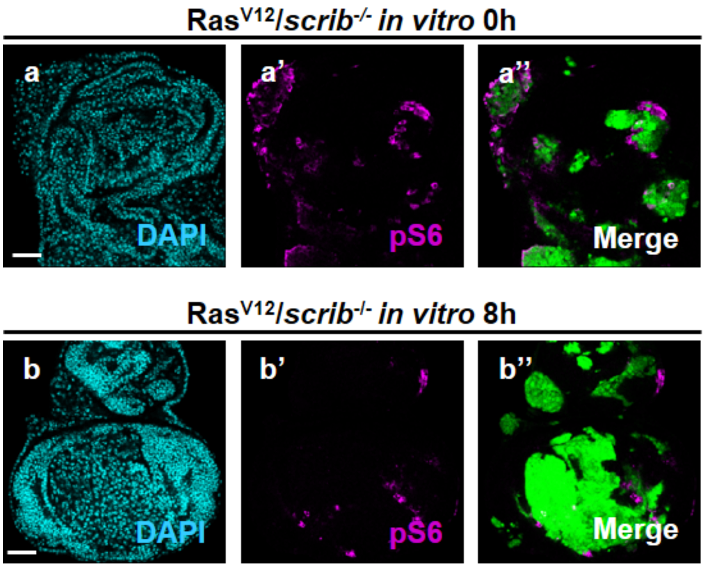

Figure R2 pS6 staining for *in vitro*-cultured Ras^{V12}/*scrib*$^{-/-}$ tumors.
(a) GFP-labeled Ras^{V12}/*scrib*$^{-/-}$ clones were induced in eye-antennal discs and stained with anti-pS6. (b) GFP-labeled Ras^{V12}/*scrib*$^{-/-}$ clones were induced in eye-antennal discs and stained with anti-pS6 after 8 hours *in vitro* culture. *In vitro* medium: 100x insulin 250μl; penicillin 250μl; FBS 500μl; Fly extract 750μl; M3 medium 25ml.
Genotype: *eyFLP1/+ or Y; Act>y+>Gal4, UAS-GFP/+; FRT82B, Tub-Gal80/UAS-Ras*^{V12}*, FRT82B, scrib*^{1} (a and b).

4. The drug effects in bantam/rab5$^{-/-}$ tumors with CG31157 overexpression should be examined.

<u>Response:</u>
We thank the reviewer for the critical comment. Following the reviewer's suggestion, we have cloned CG31157 and generated UAS-CG31157 transgenic flies, and then tested the drug effects on bantam/*rab5*$^{-/-}$ tumors with CG31157 overexpression. As a result, we found interestingly that bantam/*rab5*$^{-/-}$ tumors overexpressing CG31157 became indeed sensitive to BCH treatment (please see revised **Figure 4j**). These data support our conclusion that *CG31157* expression is critical for cells to acquire sensitivity to LAT1 inhibitors. We have now included these new data in the revised manuscript as follows:

(page 9)

"Furthermore, bantam/*rab5*$^{-/-}$ tumors overexpressing CG31157 transgene became sensitive to BCH treatment (Figure 4j), indicating a critical role of CG31157 in drug responsiveness."

(page 11, Materials and methods)

"CG31157 was PCR-amplified from cDNA of w^{1118} adult flies using primers designed to append restriction sites for enzymes EcoR1 and XbaI to the 5' and 3' end of the product. Sequence of the products were confirmed and then the product was cloned into the pUAST vector. Transgenic flies were generated by WellGenetics."

Minor comments:

5. The mechanism(s) whereby JhI-21 expression is induced by JNK plus yki is quite interesting. However, the inducers for JNK (Eiger) and yki (yki.S168A) signaling pathways are weak ones and reflect neither signaling activations and progression in two tumors. Please used HepCA and yki.S111A.S168A.S250A. Otherwise, they can not conclude "JNK or yki activation alone did not induce JhI-21 expression".

Response:

We agree with the reviewer that JNK (Eiger) or Yki (Yki.S168A) signaling activation is relatively weak. Following the reviewer's suggestion, we have now performed JhI-21 staining for Hep^{CA} or $Yki^{.S111A.S168A.S250A}$-overexpressing clones in the eye-antennal discs. As shown below (Figure R3 for the reviewer), these clones did not upregulate JhI-21. These data further confirmed our conclusion that JNK or Yki activation alone does not induce JhI-21 expression.

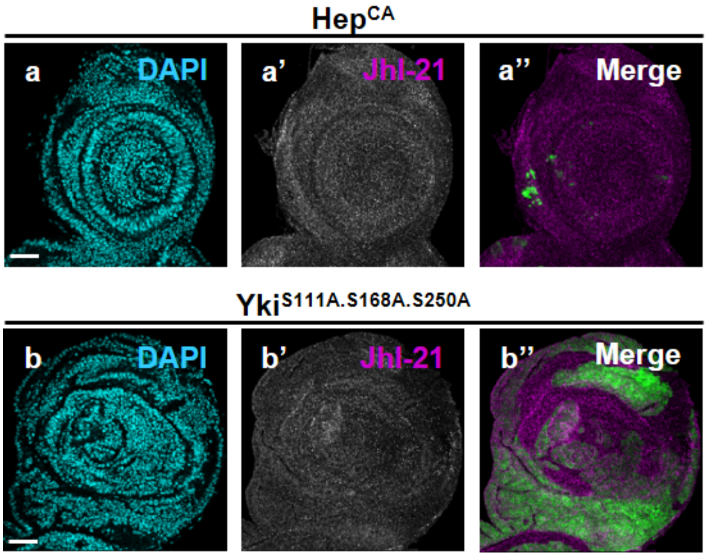

Figure R3 JhI-21 is not upregulated in Hep^{CA} or $Yki^{.S111A.S168A.S250A}$ clones.

(a) GFP-labeled Hep^{CA} clones were induced in eye-antennal discs and stained with anti-JhI-21.

(b) GFP-labeled Yki$^{S111A.S168A.S250A}$ clones were induced in eye-antennal discs and stained with anti-JhI-21.

Genotype: *eyFLP1/+ or Y; FRT40A, Tub-Gal80/FRT40A; Act>y+>Gal4, UAS-GFP/UAS-Hep*^{CA} (a), *eyFLP1/+ or Y; FRT40A, Tub-Gal80/FRT40A; Act>y+>Gal4, UAS-GFP/UAS-*yki$^{S111A.S168A.S250A}$ (b).

6. The relationships between CD98C/CG2791, Mnd, and JhI-21 have been studied to elucidate the functional regulation by heterodimeric complexes in leucine exchange in Drosophila. The authors should include them in both introduction and discussion sections to offer a general understanding of LAT physiology.

Response:
Following the reviewer's suggestion, we have now added information about CD98C/CG2791, Mnd, and JhI-21 in the introduction and discussion sections in the revised manuscript as follows:

(page 4)
"LAT1 is a plasma membrane transporter for branched-chain amino acids (BCAAs) such as leucine and isoleucine[1], thereby promoting tumor growth by activating mTOR signaling[2]. LAT1 acts as a protein complex composed of LAT1 covalently bound to 4F2 Heavy Chain Antigen (CD98/SLC3A2)[3]. CD98 promotes LAT1 protein stability and mediates the translocation of LAT1 to the cell membrane[3]."

(page 9)
"We also found that *mnd* was commonly upregulated in Ras^{V12}/*scrib*$^{-/-}$ and bantam/*rab5*$^{-/-}$ tumors and knockdown of *mnd* suppressed tumor growth. Mnd is an amino acid transporter belonging to LAT1 family, which catalyzes the cross-membrane flux of large neutral amino acids by forming heterodimers with CD98[33],[34],[35]. Thus, our data suggest that, similar to mammalian cancers, elevation of LAT1 activity is critical for tumor growth and progression in *Drosophila*. This indicates that future studies on the common oncogenesis pathway in *Drosophila* could provide novel therapeutic strategies against human cancer."

Reviewer #2: (Comments to the Author):

Igaki and colleagues use the power of Drosophila genetics and two different neoplastic tumor models to identify a role of JhI-21, a fly homolog of LAT1, a Leucine transporter, in tumor growth and malignancy. Authors use two different model systems of epithelial malignant transformation (first, the combination of an activated form of Ras: RasV12

and loss or apical-basal polarity determinants and, second, overexpression of a dominant negative form of Rab5 and overexpression of the bantam miRNA) to search, through transcriptional genomics, for genes upregulated in the two different conditions and whose depletion affetcs tumor growth. Authors make use of genetics and chemical inhibitors to unravel a role of LAT1 in tumor growth by promoting TOR signaling. Interestingly, authors identify a role of bantam miRNA in targeting CG31157, which encodes a TMEM135-like protein, and inducing resistance of these tumors to LAT1 inhibitors. The ms is well written, figures self-explanatory and the finding of LAT1 as a gene upregulated in fly tumors with a role in tumor growth will certainly contribute to the understanding of the role of the upregulation of this gene in human cancer. The identification CG31157 will also help identifying vulnerabilities of these tumors too. I have, though, two major concerns on the paper that should be addressed by the authors:

Major points:
1. Antibody against JhI-21 should be validated in overexpression experiments (in tissues overexpressing Jlh-21 with the GAL4/UAS system). It was not validated in the original paper either. Authors should show by other means the upregulation of Jhl-21 in malignant cells (eg. in situ hybridization or GFP reporters?)

Response:
We thank the reviewer for the comment. Following the reviewer's suggestion, we tried to validate the anti-JhI-21 antibody in two ways. Firstly, as indicated by the reviewer, JhI-2 was overexpressed using the FlyORF UAS-JhI-21.ORF.3xHA.GW transgene with the GAL4/UAS system and stained with the antibody. However, we could not detect upregulation of the immunostaining, probably due to the fact that LAT1 acts as a protein complex composed of LAT1 covalently bound to 4F2 Heavy Chain Antigen (CD98/SLC3A2), which promotes LAT1 protein stability and mediates the translocation of LAT1 to the cell membrane. Therefore, we have validated the antibody by detecting the upregulation of endogenous JhI-21 protein when knocking down JhI-21. As shown below (**Figure R4** for the reviewer), upregulation of anti-JhI-21 staining in Ras^{V12}/*scrib*$^{-/-}$ clones was abolished by coexpression of JhI-21-RNAi, indicating that the antibody staining is indeed working.

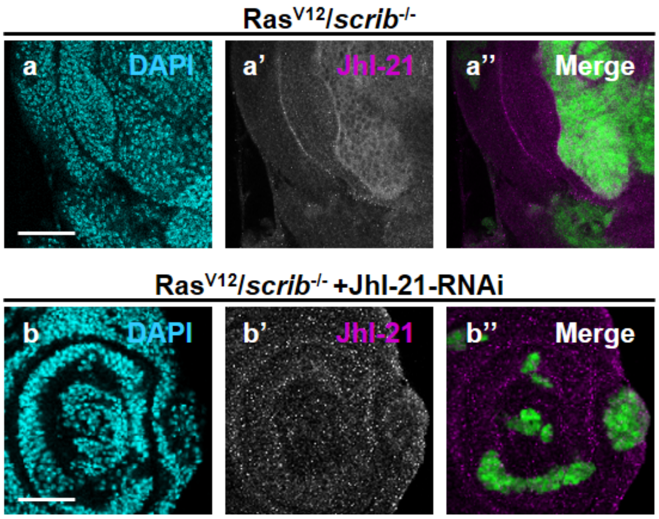

Figure R4 Validation of anti-JhI-21 antibody staining.
(a) GFP-labeled Ras^{V12}/*scrib*$^{-/-}$ clones were induced in eye-antennal discs and stained with anti-JhI-21. (b) GFP-labeled Ras^{V12}/*scrib*$^{-/-}$ +JhI-21-RNAi (HMS02271) clones were induced in eye-antennal discs and stained with anti-JhI-21.
Genotype: *eyFLP1/+ or Y; Act>y+>Gal4, UAS-GFP/+; FRT82B, Tub-Gal80/UAS-Ras^{V12}, FRT82B, scrib^{1}* (a), *eyFLP1/+ or Y; Act>y+>Gal4, UAS-GFP/UAS-JhI-21-RNAi; FRT82B, Tub-Gal80/UAS-Ras^{V12}, FRT82B, scrib^{1}* (b).

As for the latter comment ("Authors should show by other means the upregulation of Jhl-21 in malignant cells"), we have performed qRT-PCR analysis and found that, consistent with the RNA-seq data, the level of JhI-21 mRNA expression was significantly upregulated in Ras^{V12}/*scrib*$^{-/-}$ cells compared to wild-type (please see revised **Supplemental Figure S2a**). We have now included these new data in the revised manuscript as follows:

(page 6)
"In addition, qRT-PCR analysis confirmed that the level of JhI-21 mRNA expression was significantly upregulated in Ras^{V12}/*scrib*$^{-/-}$ cells compared to wild-type (Supplementary Figure 2a)."

(page 24)
"Supplementary Quantitative RT-PCR
Third instar larvae were dissected in PBS and eye-antennal discs were collected in PBS. RNA from 20~30 discs per genotype was isolated using NucleoSpin RNA XS Kit (TaKaRa). RNA samples were reverse transcribed using the SuperScript® IV reverse transcriptase (Thermo Fisher Scientific). cDNA samples were prepared by SYBR Green reagent (TOYOBO). Real-time PCR was performed using The StepOnePlus Real-Time PCR System. Primers: *JhI-21* (TTGTTTACCACGGCGAAATAG/ CTTTGTGACGGAGGAGCTACA); control gene: *RpL32* (CCAAGATCGTGAAGAAGCG/ GTTGGGCATCAGATACTGTC)."

2. JhI-21 knockdown affects the growth of the clones. I thus wonder whether the rescue of the growth of malignant clones could be a simple consequence of JhI-21 being required for cell survival or growth of normal cells. Have authors tried to use other JhI-21 RNAis not affecting the growth of control clones?

Response:
We thank the reviewer for the comment. We have analyzed another JhI-21-RNAi fly line (KK112996) and found fortunately that it did not affect the growth of normal cells (please see revised **Figure 1o**). Importantly, this JhI-21-RNAi still suppressed the growth of Ras^{V12}/*scrib*$^{-/-}$ clones (**Figure 1o**), while it did not suppress growth in wild-

type cells (please see revised Figure 1o). We have now included these new data in the revised manuscript as follows:

(page 6)
"The strongest effect was observed by JhI-21 knockdown (using two different RNAi lines HMS02271 and KK112996; Fig. 1o)."

Minor points:
3. Activation of the TOR pathway should be validated in western blots

Response:
Following the reviewer's suggestion, we have now performed a Western blot analysis to examine the activation of the TOR pathway. We found that knockdown of JhI-21 strongly suppressed the upregulation of p4EBP (an indicator of the TOR signaling activation) in Ras^{V12}/*scrib*$^{-/-}$ tumors (please see revised **Figure 3g**, quantified in **Supplementary Figure S3h**). We have now included these new data in the revised manuscript as follows:

(page 8)
"Western blot analysis for the phosphorylation of the eukaryotic initiation factor 4E binding protein (4EBP), an indicator of mTOR activation[27], confirmed that JhI-21 knockdown blocked mTOR signaling activation in Ras^{V12}/*scrib*$^{-/-}$ tumors (Fig. 3g, quantified in Supplementary Figure S3h)."

(page 12, Materials and methods)
Western blot analysis
The third instar larvae were dissected in 1x PBS and eye-antennal discs (35~40) were put into 2x sample buffer (4% SDS, 20% glycerol, 0.125M Tris-HCl (pH 6.8), 0.005% BPB, 12% 2-mercaptoethanol). After homogenization at 100°C for 5 min, samples were centrifuged at 13,000 rpm for 10 min. Lysates for the Western blot analysis were made by the supernatant. Primary antibodies used are as follows: p-4E-BP1 (Thr37/46, 1:500 in 5%BSA/TBST (0.1% Tween20 in TBS), rabbit mAb, cell signaling #2855S), monoclonal anti-α-Tubulin (1:2000 in Can get signal solution1, SIGMA T5168-2ML). Secondary antibodies used are as follows: anti-mouse IgG, HRP-linked antibody (1:10000 in Can get signal solution2, cell signaling #7076S). Images were taken with a LAS-4000 GE.

4. Code numbers in fly stocks should be included.

Response:
We thank the reviewer for the comment. We have now added code numbers of the fly stocks in the revised manuscript as follows:

(page 11)
"UAS-Ras^{V12} (BL4847, BL5788), UAS-bantam (BL60672), UAS-JhI-21-RNAi (BL41706), UAS-Rab5^{DN} (BL42704), UAS-Yki^{S168A} (BL28818), UAS-Rheb-RNAi (BL33966), *rab5*^{LL00467} (DGRC, Kyoto Institute of Technology), UAS-JhI-21-RNAi (VDRC108509), UAS-CG31157-RNAi (VDRC30296), UAS-Bsk^{DN} (T. Adachi-Yamada), *scrib*^{1} (D. Bilder)[34], UAS-Eiger^{W}, UAS-Eiger^{12} (M. Miura), *dlg*^{m52} (N. Perrimon), UAS-Wts (T. Xu)."

(page 22, Supplementary Fly strains and genetics)
"UAS-Ras^{V12} (BL4847, BL5788), UAS-bantam (BL60672), UAS-JhI-21-RNAi (BL41706), UAS-mnd-RNAi (BL62207), UAS-Rab5^{DN} (BL42704), UAS-Cam-RNAi (BL34609), UAS-cac-RNAi (4236R-1, NIG-FLY), *rab5*^{LL00467} (DGRC), UAS-CG31157-RNAi (VDRC30296, VDRC102256), *scrib*^{1} (D. Bilder), *dlg*^{m52} (N. Perrimon), UAS-Bsk^{DN} (Takashi Adachi-Yamada)."

5. A section on statistics should be added to M&M.

Response:
We thank the reviewer for the comment. We have now added a section on statistics to M&M in the revised manuscript as follows:

(page 12)
"Statistical analysis
GFP marked clones were measured by ImageJ. Statistical significances were calculated using Student's t-test, *P<0.05, **P<0.01 and ***P<0.001. Graphs were made by ggplot2."

6. A wild type eye should be added to Figure 1 for comparison.

Response:
Following the reviewer's suggestion, we have now added a wild-type adult eye in Figure 1 as a control (please see revised **Figure 1i**) in the revised manuscript as follows:

(page 6)
"and rescued lethality of animals bearing these tumors (Fig.1k, compare to 1h and 1i"

Reviewer #3: (Comments to the Author):

This manuscript explores the control of cell growth in Drosophila neoplastic tumours. A strong upregulation of the TOR pathway is identified by pS6 staining, and this is attributed to a very mild upregulation of LAT1 amino-acid transporter.

The basic problem is that the magnitude of the induction of LAT1 is very, very small compared with the magnitude of the pS6 induction, which means that the authors have not identified the actual mechanism primarily responsible for inducing TOR activation in these tumours. Thus, I cannot support publication.

Response:

We agree with the reviewer that the magnitude of the induction of LAT1 is small compared with the magnitude of the pS6 induction. However, this does not conflict with our conclusion. We found that knockdown of LAT1 in tumors strongly blocked TOR signaling activation (by examining both pS6 immunostaining and p4EBP Western blotting). These data suggest that small upregulation of LAT1 significantly activates TOR signaling in tumors. As supported by other reviewers, we believe that our data presented in this manuscript identify the mechanism primarily responsible for TOR activation in these tumors.

Reviewer #4: (Comments to the Author):

Cong and colleagues investigate tissue growth control in Drosophila imaginal discs. They use several well studied genetic combinations (RAS, Hippo, scribble, Rab5, bantam) that perturb cell polarity and proliferation. The power of the study is the unbiased RNA-sequencing experiment and subsequent mini genetic screen where they identify genes that can partially reverse the epithelia tumor overgrowth that is caused by perturbing these factors. They identify the Juvenile hormone Inducible-21 (JhI-21), a Drosophila homolog of the L-amino acid transporter 1 (LAT1), as a gene that is elevated in the tumors and required for their growth. They propose that JNK and the Hippo protein Yorkie drive its expression and that Jhl-21 induces the Tor pathway to drive tumor growth. They further explore the role of the microRNA bantam in these tumors.

The study is interesting and the screen is powerful. They use many approaches and experiments that they and others have employed in other studies, and often repeat published results (e.g. warts expression can suppress RAS/scribble tumors). They have some interesting observations that specialists within the Drosophila tumor field will appreciate. At this stage I don’t think the manuscript will be of substantial interest to non-specialists. The major shortfall of the study is that the mechanism they have out

forward for how the different factors intersect (Hippo, polarity, Jhl-21, Tor etc. is not well substantiated and hard to reconcile. The paper reads more like a conglomeration of interesting observations than a pithy dissection/discovery of a new feature of epithelial tissue growth. However, I think the paper will help to shape the studies of others in the fly epithelial tissue growth field, although the manuscript requires far more effort to properly present and discuss the data. In particular, the authors to validate their Jhl-21 experiments by using independent RNAi line and/or mutants and provide validation of the Jhl-21 antibody. Comments that should help to improve the manuscript are:

Introduction:

1. This needs more text to properly introduce the study. Also, it jumps too abruptly from para 1 to para 2. Given that LAT1 was found in an unbiased fashion it is probably better introduced at the end of the introduction. Similarly, the Discussion is very brief and does not adequately discuss the findings of the study, their significance and limitations and important areas to address in the future.

Response:

We thank the reviewer for the comment. Following the reviewer's suggestion, we have now revised the introduction and discussion sections in the revised manuscript as follows:

(page 4)

"In recent years, amino acid transporter, especially L-amino acid transporter 1 (LAT1), has been attracting attention as a potential therapeutic target for cancer. LAT1 is a plasma membrane transporter for branched-chain amino acids (BCAAs) such as leucine and isoleucine[1], thereby promoting tumor growth by activating mTOR signaling[2]. LAT1 acts as a protein complex composed of LAT1 covalently bound to 4F2 Heavy Chain Antigen (CD98/SLC3A2)[3]. CD98 promotes LAT1 protein stability and mediates the translocation of LAT1 to the cell membrane[3]. Studies in mammalian cells have shown that LAT1 is upregulated in neuroblastoma and Burkitt's lymphoma cells via Myc[4] and in breast cancer cells via aryl hydrocarbon receptor (AHR)-mediated signaling[5]. Given that LAT1 expression is elevated in a variety of cancers, it is thought to be an ideal therapeutic target as a component of a common oncogenesis pathway and is thus currently under clinical trial in cancer patients[2],[6]."

"Here, through a transcriptome analysis combined with *Drosophila* genetics, we searched for a common pathway of oncogenesis among different types of *Drosophila* malignant tumors. By an unbiased transcriptome analysis, we found that LAT1 expression was commonly elevated in *Drosophila* malignant tumors with different oncogenic mutations."

(page 9)

"We also found that *mnd* was commonly upregulated in Ras^{V12}/*scrib*$^{-/-}$ and bantam/*rab5*$^{-/-}$ tumors and knockdown of *mnd* suppressed tumor growth. Mnd is an amino acid transporter belonging to LAT1 family, which catalyzes the cross-membrane flux of large neutral amino acids by forming heterodimers with CD98[33],[34],[35]. Thus, our data suggest that, similar to mammalian cancers, elevation of LAT1 activity is critical for tumor growth and progression in *Drosophila*. This indicates that future studies on the common oncogenesis pathway in *Drosophila* could provide novel therapeutic strategies against human cancer. Indeed, our data intriguingly show that overexpression of a pro-growth microRNA bantam rendered malignant tumors resistant to LAT1 inhibitory drugs."

(page 10)

"Thus, our observations not only show an evolutionarily conserved role of LAT1 induction in driving tumor malignancy but provide a useful genetic model for studying the mechanism of drug resistance."

2. The Jhl-21 RNAi line used should be verified with protein expression and/or RNA experiments, to prove that it targets Jhl-21 expression. They can verify the Jhl-21 antibody in imaginal discs and the RNAi line in one experiment. At least one other Jhl-21 RNAi line should be used to verify the experiments presented in this paper. Alternatively, a mutant allele should be used.

Response:

We thank the reviewer for the comment. As for the validation of the Jhl-21 RNAi (HMS02271), we have performed anti-JhI-21 staining in Ras^{V12}/*scrib*$^{-/-}$ clones with or without Jhl-21 RNAi expression. As shown below (**Figure R5** for the reviewer, below), upregulation of anti-JhI-21 staining in Ras^{V12}/*scrib*$^{-/-}$ clones was completely abolished by coexpression of JhI-21-RNAi, which validated the effect of Jhl-21 RNAi.

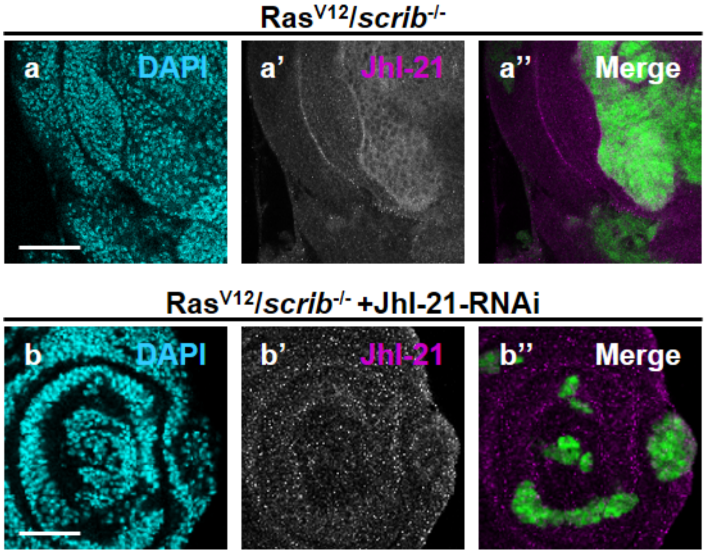

Figure R5 Validation of the JhI-21-RNAi line.
(a) GFP-labeled Ras^{V12}/*scrib*$^{-/-}$ clones were induced in eye-antennal discs and stained with anti-JhI-21. (b) GFP-labeled Ras^{V12}/*scrib*$^{-/-}$ +JhI-21-RNAi (HMS02271) clones were induced in eye-antennal discs and stained with anti-JhI-21.
Genotype: *eyFLP1/+ or Y; Act>y+>Gal4, UAS-GFP/+; FRT82B, Tub-Gal80/UAS-Ras^{V12}, FRT82B, scrib^{1}* (a), *eyFLP1/+ or Y; Act>y+>Gal4, UAS-GFP/UAS-JhI-21-RNAi; FRT82B, Tub-Gal80/UAS-Ras^{V12}, FRT82B, scrib^{1}* (b).

In addition, following the reviewer's suggestion, we have now performed additional experiments using a different JhI-21-RNAi line (VDRC KK112996) and found that it also significantly suppressed growth of Ras^{V12}/*scrib*$^{-/-}$ tumors while it did not affect the growth of normal clones (please see revised **Figure 1o**). We have now included these new data in the revised manuscript as follows:

(page 6)
"The strongest effect was observed by JhI-21 knockdown (using two different RNAi lines HMS02271 and KK112996; Fig. 1o)."

3. Rheb mutant alleles impair cell growth and proliferation. Why does Rheb RNAi give no phenotype in Figure 3. Has it been proven to deplete Rheb levels? If not, this should be done.

<u>Response:</u>
We thank the reviewer for the comment. Following the reviewer's suggestion, we have performed pS6 staining of eye-antennal imaginal discs bearing Rheb-RNAi clones to validate the effect of Rheb-RNAi. In wild-type eye-antennal discs, pS6 is upregulated around the morphogenetic furrow (please see Figure R6a, below). This pS6 staining was abolished in Rheb-RNAi clones at the morphogenetic furrow (Figure R5b, arrowheads), indicating that the Rheb-RNAi indeed suppresses TOR signaling.

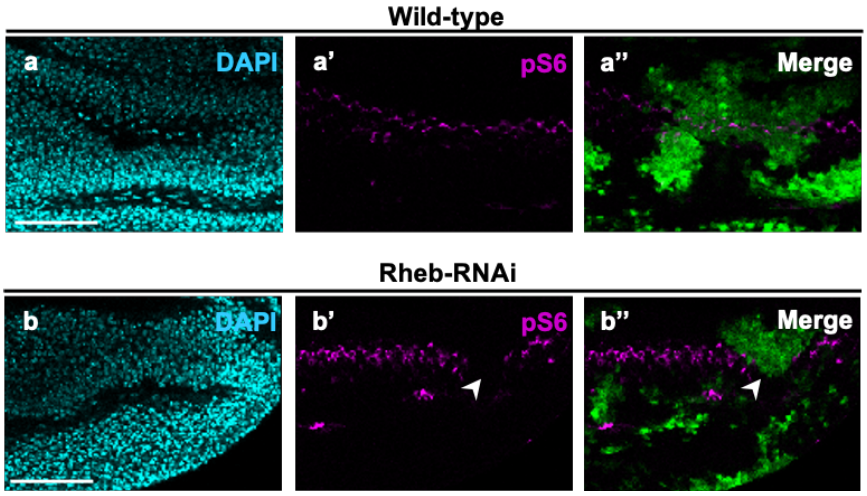

Figure R6 Validation of the Rheb-RNAi line.

(a) GFP-labeled wild-type clones were induced in eye-antennal discs and stained with anti-pS6.

(b) GFP-labeled Rheb-RNAi clones were induced in eye-antennal discs and stained with anti-pS6.

Genotype: *eyFLP1/+ or Y; Act>y+>Gal4, UAS-GFP/+; FRT82B, Tub-Gal80/FRT82B* (a), *FRT19A, Tub-Gal80/FRT19A; eyFRP5, Act>y+>Gal4, UAS-GFP/+; Rheb-RNAi/sb* (b).
