# Supplementary material for: JNK and Yorkie drive tumor malignancy by inducing L-amino acid transporter 1 in Drosophila
Source: PLoS Genet. 2021 Nov 15;17(11):e1009893. doi: 10.1371/journal.pgen.1009893 (PMC8629376; doi:10.1371/journal.pgen.1009893)
Supplement: Attachment — Submitted filename: Responses to Reviewers.pdf [file pgen.1009893.s011.pdf]

Here we describe our second round of revisions and responses to reviewer's comments. We would like to thank the reviewers for excellent comments, which have led us to improve our manuscript further. Modifications are indicated in red in the revised manuscript.

Response to Reviewers' Comments:

Reviewer #1 and #4 raised no further concerns on our previous revised manuscript. We therefore describe our responses to Reviewer #2 and #3.

Reviewer #2: (Comments to the Author):

Authors have addressed satisfactorily some of my previous concerns. I still have two major issues:

(1) I do believe authors should take statistics more seriously. First, Student t-test statistical analysis is not correct in most cases and authors should use alternative ones such as Dunnet's due to the presence of more than one experimental set. Second, authors should include a supplementary table where all data are summarised: n, p values, statistical analysis used, mean, standard deviation and p values for all figure panels where a quantification is being done. Third, authors should explicitly explain which program was used to perform the statistical analysis and say whether all experiments were done the same time.

Response:

(First to third) We thank the reviewer for the comments. The reviewer is right and thus we have re-analyzed all the data using appropriate statistical methods such as Dunnet's multiple comparison test, Tukey's multiple comparison test, and Mann Whitney test. We have now included Supplementary Table S3 that describes all the statistical information (n, p values, statistical methods and software used, mean, and standard deviation for all figure panels) in the revised manuscript. We also explained the program used in the statistical analysis and mentioned that all experiments were done at the same time.

(Page 12)

"Statistical analysis

The areas of GFP-labeled clones were measured by ImageJ. Statistical analysis was performed using Prims9. All experiments shown in the same graph were done at the same time. N.S P(>0.12), * P(0.033), ** P(0.002) and *** P<0.001. All statistical data were summarized in Supplementary Table S3."

Fourth, I wonder whether the same Ras/scrib control was used to analyse the impact of two RNAis to rescue clone size in Figure 1o. I hope the answer is no. If so, two different control bars should be included or the second set of experiments where RAS/scrib clone

size was compared to the new set of clones in the presence of the new RNAi.

Response:

Actually, we had performed the RNAi experiments at the same time; we originally used two different JhI-21 RNAi lines to test whether they could suppress Ras/scrib overgrowth and, given that both of them suppressed the overgrowth, we used only one RNAi data (more effective one) in the manuscript. To respond the reviewer's concern that multiple RNAi lines should be tested, we have added the other RNAi data in the same graph in the revised manuscript. Therefore, we used a single control data for both RNAi data in Figure 1o.

(2) I am not convinced with the answer to my concern on the antibody against JhI-21. Have authors tested whether the protein can be detected with an antibody to HA: when the UAS-JhI-21.ORF.3xHA.GW transgene is over expressed with the GAL4/UAS system? There are EP-lines available. Can authors test in this case?

Response:

We thank the reviewer for the comment. Following the reviewer's suggestion, we have performed anti-HA staining of eye discs bearing clones of cells overexpressing UAS-JhI-21.ORF.3xHA.GW. As a result, we found that anti-HA staining was detected within the clones but anti-JhI-21 staining was not detected (please see Figure R1c, below). Importantly, these clones did not upregulate anti-pS6 staining either (Figure R1d, below), indicating that the product of this transgene does not activate TOR signaling. We also used an EP line (UAS-JhI-21[GE15185]), which also detected neither anti-JhI-21 nor anti-pS6 staining (Figure R1e and R1f, below). As we described in the first round of revision, LAT1 acts as a protein complex with 4F2 Heavy Chain Antigen (CD98/SLC3A2), which promotes LAT1 protein stability and mediates the translocation of LAT1 to the cell membrane where it is functional. Thus, our data suggest that transgene-mediated overexpression of JhI-21 alone is somehow non-functional or even dominant-negative (as clones are very small) and cannot be detected by anti-JhI-21 staining. Given that anti-JhI-21 staining constantly detect its upregulation in Ras^{V12}/*scrib*$^{-/-}$ clones and this is canceled by JhI-21-RNAi (Figure R1a and R1b, below), these data strongly suggest that the antibody detects endogenous upregulation of the functional JhI-21 protein. Since this is an intriguing observation, we would like to further investigate how JhI-21 protein functions in tumors in our future study.

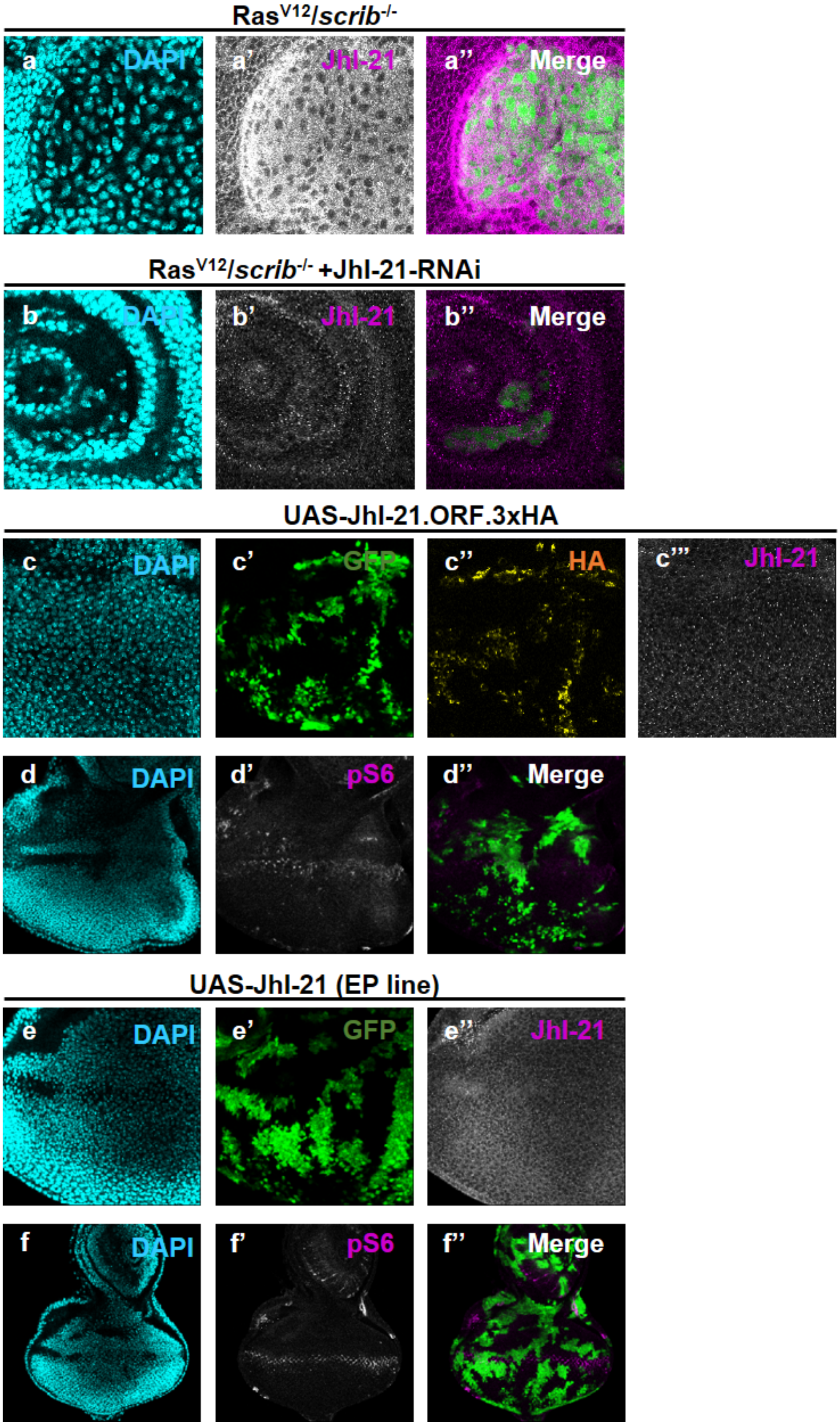

Figure R1: Validation of JhI-21 antibody.

(a, b, c, d, e and f) GFP-labeled Ras^{V12}/*scrib*$^{-/-}$ (a), Ras^{V12}/*scrib*$^{-/-}$ +JhI-21-RNAi ^{HMS02271} (b), UAS-JhI-21.ORF.3xHA (c and d), or UAS-JhI-21^{GE15185} (e and f) clones were induced in eye-antennal discs and stained with anti-JhI-21, anti-HA or pS6.

Genotype: *eyFLP1/+ or Y; Act>y+>Gal4, UAS-GFP/+; FRT82B, Tub-Gal80/UAS-Ras*^{V12}*, FRT82B, scrib*^{1} (a), *eyFLP1/+ or Y; Act>y+>Gal4, UAS-GFP/UAS-JhI-21-RNAi; FRT82B,*

Tub-Gal80/UAS-Ras^{V12}, FRT82B, scrib^{1} (b), *eyFLP1/+ or Y; FRT40A, Tub-Gal80/FRT40A; Act>y+>Gal4, UAS-GFP/UAS-JhI-21.ORF.3xHA* (c and d). *eyFLP1/+ or Y; Act>y+>Gal4, UAS-GFP/ UAS-JhI-21^{GE15185}; FRT82B, Tub-Gal80/ FRT82B* (e and f).

Reviewer #3: (Comments to the Author):

The authors still didn't address the basic problem. It is no surprise that you need amino acid transport for TOR activation. The problem is that they attribute the strongly increased TOR activation in their tumours to a very mild upregulation of LAT1, which is just not a well supported conclusion.

Response:

As pointed out by the reviewer, one of the most interesting observations in this study is that relatively "mild" upregulation (3.7-fold transcriptional upregulation compared to wild-type) of JhI-21/LAT1 can be the cause of strong activation of TOR signaling in Ras^{V12}/*scrib* tumors. However, it is even possible that the upregulation of JhI-21/LAT1 is not "mild", as we cannot evaluate the upregulation of "functional LAT1 protein" at this stage. LAT1 acts as a protein complex with 4F2 Heavy Chain Antigen (CD98/SLC3A2), which promotes LAT1 protein stability and mediates the translocation of LAT1 to the cell membrane where it is functional. It is possible that Ras^{V12}/*scrib* tumors possess yet unidentified mechanism that efficiently utilize upregulated JhI-21/LAT1 as "functional LAT1 protein". This study identified that Ras^{V12}/*scrib* tumors strongly upregulate TOR signaling, which is essential for their growth, and that LAT1 is "mildly" upregulated in Ras^{V12}/*scrib* tumors and LAT1 inhibitors significantly inhibit growth of Ras^{V12}/*scrib* tumors while they do not affect growth of wild-type tissue. We believe that all these observations are critical and novel, support our conclusions, and add a significant progress in this field.

Our RNA-seq data showed that expression of JhI-21/LAT1 was upregulated ~3.7-fold in Ras^{V12}/*scrib*$^{-/-}$ cells compared to wild-type. Interestingly, a recent study in mice showed that LAT1 is upregulated ~1.5-fold in colon tumors and ~10-fold in small intestinal tumors, which was shown to be the cause of mTOR activation in tumor cells (please see Figure R2 cited from Najumudeen et al., *Nat Genet*., 2021, below, which came out after we submitted our manuscript to PLOS Genetics). These data are very similar to ours and suggest that the role of LAT1 upregulation in tumorigenesis could be evolutionarily conserved.

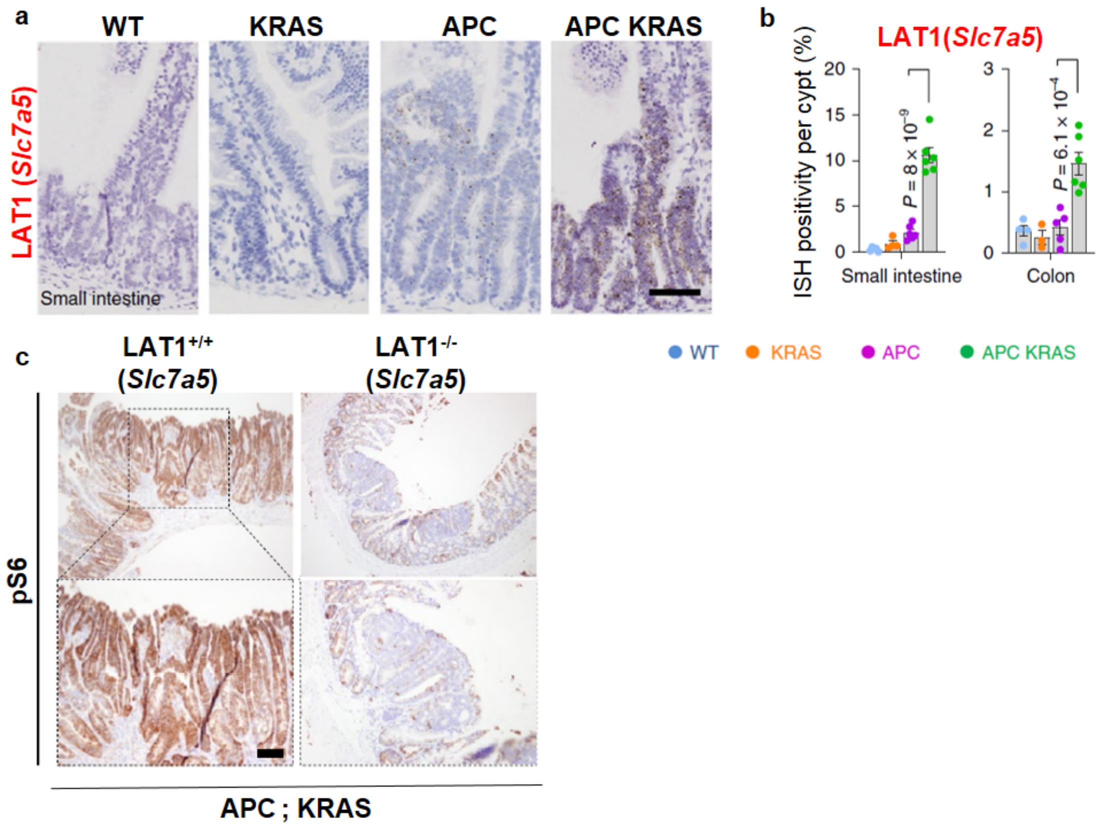

Figure R2: *SLC7A5* (LAT1) loss suppresses mTOR signaling (cited from Najumudeen et al., *Nat Genet*., 2021).

(a) Representative images showing *Slc7a5* expression in small intestines from WT, KRAS, APC and APC KRAS mice detected by RNA in situ hybridization (ISH). Scale bars, 200 µm. (b) Quantification of *Slc7a5* ISH staining in the small intestine and colon. Small intestine (WT, KRAS, n = 4; APC, n = 6; APC KRAS, n = 6), colon (WT, n = 4; KRAS, n = 3; APC, n = 5; 25 half-crypts were scored per mouse. Data are mean ± s.e.m. P = 8 × 10−9 (small intestine), P = 6.1 × 10−4 (colon), using one-way ANOVA followed by Tukey's test. (c) Immunohistochemistry (IHC) for phosphorylated RPS6 (pS6) in small intestinal tumors of the indicated genotypes (*Apc*$^{fl/fl}$; *Kras*$^{G12D/+}$; *Slc7a5*$^{+/+}$ and *Apc*$^{fl/fl}$; *Kras*$^{G12D/+}$; *Slc7a5*$^{fl/fl}$ mice).
